# Eyes of Fibromyalgia

**DOI:** 10.3390/biomedicines14081656

**Published:** 2026-07-23

**Authors:** Arzu Dinç Yavaş, Akasya Şaipi, Sümeyye Akçay

**Affiliations:** 1 Department of Physical Medicine and Rehabilitation, Faculty of Medicine, Istanbul Aydın University, 34295 Istanbul, Türkiye; arzudinc0111@gmail.com; 2 Department of Ophthalmology, Faculty of Medicine, Istanbul Medipol University, 34810 Istanbul, Türkiye; akasya.saipi@yandex.com; 3 Department of Occupational Therapy, Faculty of Healthy Sciences, Fenerbahçe University, 34758 Istanbul, Türkiye

**Keywords:** retina, fibromyalgia, dry eye

## Abstract

**Background/Objectives**: Fibromyalgia (FM) is a multifactorial disorder characterized by chronic pain, fatigue, asthenia, and sleep disturbances. FM may have effects on the eyes, such as dry eye disease (DED) and retinal changes. The aim of this study is to evaluate these ocular changes in FM patients. **Methods**: It is a single-center prospective study. Forty-two female patients with FM (Group 1) and 37 healthy controls (Group 2) participated in the study. Study participants underwent screening tests for DED and the retinal nerve fiber layer (RNFL) thickness. **Results**: According to the Schirmer test, Group 1 had lower scores with a significant difference (8.2 ± 4.36 mm vs. 17.51 ± 2.41 mm). When the tear film breakup time (TBUT) test values were analyzed, there was a significant difference between groups; Group 1 had lower scores (4.95 ± 2.26 s vs. 9.86 ± 1.64 s). When the RNFL thickness values were analyzed by quadrants, our results in Group 1 FM patients were lower in the superior temporal quadrant (117.98 ± 13.98 µm vs. 124.35 ± 13.71 µm). **Conclusions**: These findings indicate that patients with fibromyalgia may have increased dry eye findings and localized RNFL thinning, suggesting that fibromyalgia may be associated with both anterior and posterior segment ocular alterations. However, these parameters should be interpreted as ocular manifestations rather than diagnostic markers. Further studies are needed to determine their clinical relevance and potential biomarker value.

## 1. Introduction

Fibromyalgia (FM) is a multifactorial disorder characterized by chronic pain, fatigue, asthenia, and sleep disturbances among other symptoms [1]. The prevalence of FM is 1–4% in the general population, and there is a higher prevalence in the adult female population (2.5–10.5%). The etiology and pathogenesis of FM are not fully understood; thus, it is thought to involve multiple factors, including central and autonomic nervous system dysfunction, changes in neurotransmitter and hormone levels, abnormal immune responses, external stressors, and psychological factors [1,2].

Neuroimaging studies have demonstrated hypoperfusion in the thalamus and caudate nucleus in FM patients, along with evidence of small nerve fiber pathology [3,4,5,6,7,8,9]. Separately, retinal nerve fiber layer (RNFL) thinning at the optic nerve head, as measured by optical coherence tomography (OCT), is a well-established finding in neurodegenerative diseases such as Parkinson’s disease, Alzheimer’s disease, neuromyelitis optica, and Multiple Sclerosis [9,10,11,12,13,14,15]. Based on these observations, it has been proposed that FM may also involve retinal neurodegeneration: decreased RNFL thickness, ocular pain, necrotizing scleritis, and altered optic disc perfusion have been reported as ocular features of FM in a limited number of studies [15,16], with RNFL thinning most consistently observed in the temporal region. Onuora et al. further reported axonal damage in the optic nerve of FM patients, suggesting—though not yet confirming—that neurodegeneration may contribute to the disease’s pathophysiology [15,16]. However, the mechanistic link between small-fiber neuropathy and retinal involvement in FM remains hypothetical and has not been directly demonstrated. Findings across these studies have also not been entirely consistent: Garcia-Martin et al. reported RNFL thinning using two different OCT platforms, with the specific quadrants reaching statistical significance differing between devices [15], while Urfalıoglu et al. linked the degree of RNFL thinning to FM severity rather than to FM diagnosis alone [17]. This variability across OCT devices and study populations highlights the need for further studies to clarify whether RNFL thinning is a consistent and reproducible ocular finding in FM.

FM patients also have a higher incidence of dry eye disease (DED) compared to the normal population [18,19]. Small fiber neuropathy and dysautonomia are considered the reasons for corneal denervation and dry eye symptoms in FM [20].

We hypothesized that female FM patients would show objectively measurable anterior segment (tear film) and posterior segment (RNFL) ocular alterations compared with healthy controls. Accordingly, this study aimed to characterize these ocular findings as exploratory, clinic-accessible correlates of the small-fiber neuropathy proposed to underlie FM, rather than as diagnostic biomarkers.

## 2. Materials and Methods

The study protocol was approved by the Medipol University Faculty of Medicine Clinical Research Ethics Committee (Date: 14 October 2022, No: E-10840098-772.02-6187). The study was conducted in accordance with the principles of the Declaration of Helsinki. Informed consent of the participants was obtained.

### 2.1. Study Design

Our single-center prospective study was conducted in Physical Medicine and Rehabilitation and Ophthalmology clinics. Female patients who were diagnosed with FM and between 18–60 years of age were included in the study (Group 1, *n* = 42). Patients with conditions that may affect the eye and vision (significant refractive errors [>5 diopters of spherical equivalent refraction or 3 diopters of astigmatism], use of contact lenses, cataract, glaucoma, retinopathy, uveitis, heart disease, hypertension, rheumatological pathologies, and neurological diseases) were excluded. Patients using medications with known anticholinergic or lacrimal-secretory effects (e.g., pregabalin, duloxetine, amitriptyline, or SSRIs/SNRIs) for indications other than FM were also excluded from both groups. The control group (Group 2, *n* = 37) consisted of healthy female volunteers of a similar age range, with no previously diagnosed rheumatological or ocular disease, recruited from the hospital’s routine check-up department. Female patients complaining of widespread pain were examined by a physical medicine and rehabilitation specialist, and patients who fulfilled the American College of Rheumatology (ACR) 2010 criteria were diagnosed as FM [21]. All FM patients were newly diagnosed and had not yet initiated pharmacological treatment for FM (e.g., pregabalin, duloxetine, amitriptyline, or SSRIs/SNRIs) at the time of ophthalmic assessment; all ocular measurements were obtained prior to the initiation of any such therapy. Patients who were diagnosed with FM were then referred to the ophthalmologist, and various ophthalmic measurements were performed.

### 2.2. Ophthalmological Examinations

A total of 42 eyes from 42 FM patients and 37 eyes from 37 healthy individuals were included in this study. Measurements were performed for both eyes, but only the right eye values were included in the statistical analyses, as paired-eye measurements from the same individual are not statistically independent; using a single eye per patient avoided violating this independence assumption. Study participants underwent a screening procedure for DED, which consisted of a Schirmer’s test (Haag-Streit UK Ltd., Harlow, Essex, UK) and tear film breakup time (TBUT) test [22].

For the Schirmer test, a 5 × 35 mm filter paper strip was left for 5 min on the lateral part of the inferior fornix. The length of the wetness on the strip was the indicator of tear fluid production. The cut-off value for defining eye dryness was based on a Schirmer’s test value of ≤5 mm within 5 min [23].

To evaluate tear film stability and quality, TBUT was measured. The cornea of the inferior fornix was touched with a fluorescein strip, and the time between the last eye blink and the first dry spot was measured. Time measured above 10 s was considered normal [24].

All measurements were performed by a single, masked examiner. The RNFL thickness of the four quadrants of the retina was measured using the OCT Spectralis^®^ (2022, Heidelberg Engineering, Heidelberg, Germany) system at a scan rate of 85,000 Hz, with an axial resolution of 3.9 μm and a scan depth of 1.9 mm, using an 880 nm light source. All measurements were obtained according to the manufacturer’s recommendations for optic nerve scan and image capture. Images with poor resolution, motion artifacts, and/or a signal strength index <50 were excluded. All measurements were automatically recorded and calculated using the AngioAnalytics software version 2018.0.0.14. As in a comparable OCT-based study in fibromyalgia [15], scan segmentation was not manually corrected; images failing to meet the quality criteria described above were excluded rather than corrected.

### 2.3. Statistical Analysis

Statistical analyses were performed using SPSS version 25.0 (IBM Corporation, Armonk, NY, USA). Histograms and the Kolmogorov-Smirnov test were used to assess the normality of the variables. Mean, standard deviation, median, and min-max values were used in descriptive analyses. Non-normally distributed (non-parametric) variables were compared between two groups using the Mann–Whitney U Test, and normally distributed (parametric) variables were compared between two groups using the Independent *t*-Test. *p*-values below 0.05 were considered statistically significant. Effect sizes (Cohen’s d) with 95% confidence intervals were calculated for RNFL quadrant comparisons between groups, and *p*-values were adjusted for multiple comparisons across the four quadrants using the Bonferroni correction.

No a priori sample size calculation was performed, as this was an exploratory study; the sample size was determined by the number of eligible patients recruited during the study period. A post hoc sensitivity analysis indicated that, with the achieved sample sizes (*n* = 42 and *n* = 37), the study had 80% power (α = 0.05, two-tailed) to detect a between-group difference of Cohen’s d ≥ 0.64 or larger; smaller effects may not have been reliably detected.

## 3. Results

There was no statistically significant difference in patients’ ages between the groups (*p* = 0.100). The mean ages of the patients were 40.81 ± 5.25 years in Group 1 and 39.03 ± 9.59 years in Group 2. When the Schirmer test and TBUT test values were analyzed, there was a significant difference between groups: Schirmer test values were 8.2 ± 4.36 mm in Group 1 and 17.51 ± 2.41 mm in Group 2 (*p* < 0.001), and TBUT values were 4.95 ± 2.26 s in Group 1 and 9.86 ± 1.64 s in Group 2 (*p* < 0.001). Table 1 summarizes the demographic and dry eye test results of the study groups.

When the RNFL thickness was analyzed by quadrants, the RNFL was thinner in all quadrants in Group 1, and a significant difference was seen in the superior temporal quadrant (117.98 ± 13.98 µm) (*p* = 0.045). Effect sizes (Cohen’s d) for the four RNFL quadrants ranged from −0.46 to 0.03, indicating small-to-moderate between-group differences (Table 2). After Bonferroni correction for the four quadrant comparisons, the superior temporal difference did not remain statistically significant (corrected *p* = 0.180), suggesting that this finding should be interpreted with caution.

Table 2 states the RNFL thickness values in all quadrants.

To further explore the anterior segment (tear film) and posterior segment (RNFL) relationship within the FM group, we examined the correlation between tear film parameters and RNFL thickness in each quadrant. No significant correlation was found between Schirmer test or TBUT values and RNFL thickness in any quadrant (Table 3).

## 4. Discussion

FM is described as a “central sensitization syndrome” resulting from neurobiological and structural brain abnormalities [4,25,26,27,28]. In FM patients, changes in cerebral blood flow, functional neural plasticity, and brain morphology are related to pain [4,15]. Cerebral imaging techniques reveal hypoperfusion in the bilateral thalamus and bilateral caudate nucleus, which are important for nociception [4]. Additionally, autonomous nervous system dysregulation resulting in regional vasomotor problems leads to muscle hypoperfusion and pain [3,29].

Retinal nerve fiber layer examination provides a window into central nervous system integrity, as RNFL thickness measured by OCT correlates well with brain MRI measurements [25,30,31,32]. There are a few studies on retinal changes in FM patients [15,16,17].

In classic neurodegenerative diseases, the temporal superior RNFL quadrant is often preferentially affected because its fibers follow the papillomacular bundle; this rationale has motivated several studies to examine this quadrant specifically in FM [15,16,17]. However, whether FM itself constitutes a neurodegenerative process remains unproven, and the present findings should be interpreted as a localized structural difference rather than definitive evidence of neurodegeneration, particularly given that only one of four quadrants reached statistical significance and disease severity was not assessed. Garcia-Martin et al. [15] reported a significant decrease in RNFL thickness in patients with FM compared with controls. They used two different OCT devices: one was the Cirrus OCT, which performed ganglion cell layer analysis. They found that FM patients have lower RNFL regional thickness values, and these changes were correlated with disease severity. A significant decrease in the RNFL was detected in fibromyalgia patients compared with controls using the two OCT devices: Cirrus OCT ganglion cell layer analysis registered a significant decrease in the minimum thickness of the inner plexiform layer (74.99 ± 16.63 μm vs. 79.36 ± 3.38 μm, respectively; *p* = 0.023), nasal inferior, temporal inferior, and temporal superior sectors (*p* = 0.040; 0.011 and 0.046, respectively). The other device was the Spectralis OCT, which revealed thinning in the nasal, inferior temporal, and superior temporal sectors (*p* = 0.009, 0.006, and 0.002, respectively) in fibromyalgia patients, and the axonal application showed thinning in all sectors except the nasal superior and temporal sectors.

In another study, researchers evaluated quantitative changes in RNFL thickness in patients with mild-to-moderate and severe FM compared with healthy individuals. Average retinal nerve fiber layer thickness values were significantly lower in severe FM (101.18 ± 6.03 mm) than in mild-moderate FM (103.21 ± 10.66 mm) and the control group (106.51 ± 8.88 mm) (*p* = 0.041 and 0.020, respectively). The inferotemporal (134.36 ± 12.19 mm) and inferonasal (109.47 ± 16.03 mm) quadrant retinal nerve fiber layer thickness values were significantly lower in severe FM than mild-moderate FM patients [inferotemporal (142.15 ± 17.79 mm), inferonasal (117.94 ± 20.53 mm)] and Group 3 [inferotemporal (144.70 ± 16.25 mm), inferonasal (118.63 ± 19.01 mm)] [inferotemporal, *p* = 0.017 and 0.010, respectively; inferonasal, *p* = 0.047 and 0.045, respectively]. The nasal-superior quadrant retinal nerve fiber layer thickness value was higher in healthy controls (91.08 ± 12.11 mm) than in mild-moderate FM (84.34 ± 13.09 mm) and severe FM (85.26 ± 13.11 mm) (*p* = 0.031 and 0.038, respectively). Lower RNFL thickness was associated with greater FM severity [17]. The RNFL thickness values in our FM patient group were as follows: inferior temporal region (123.67 ± 15.51 mm), superior temporal (117.98 ± 13.98 mm), inferior nasal (72.36 ± 9.81 mm), and superior nasal (65.4 ± 11.92 mm). In our study, RNFL thickness was nominally lower in the superior temporal quadrant (uncorrected *p* = 0.045; this difference did not remain significant after Bonferroni correction for the four quadrants, see Table 2), broadly consistent with the pattern reported in previous studies [15,16,17,33]. We did not stratify patients by FM severity, and the discrepancies with previous studies may be attributable to differences in FM severity distribution as well as the OCT device used.

In previous studies, DED in FM was investigated using the Schirmer’s test, and the reported prevalence ranged from 32% to 90% [34,35,36,37]. Dry eye is common among patients with chronic musculoskeletal pain, with and without an FM diagnosis [38]. FM patients are reported to have a higher rate of DED (76%). In a recent study, researchers investigated dry eye in FM patients using Schirmer and TBUT tests. Both Schirmer (16.1 mm vs. 15.3 mm, *p* = 0.36) and TBUT (17.8 s vs. 18.8 s, *p* = 0.40) results were similar in the FM group and control subjects [39]. Unlike this study, our results supported earlier findings that patients with FM have eye dryness, as measured by the Schirmer test (8.2 ± 4.36 mm, *p* < 0.001) and the TBUT test (4.95 ± 2.26 s, *p* < 0.001). These differences in the literature may be due to testing procedures, such as whether topical anesthesia was used.

Impairment of polymodal nociceptor activity and reduction of corneal sensitivity lead to DED in FM patients. FM is described as a type of altered central sensitivity syndrome. Corneal nerve fiber density is negatively correlated with peripheral and central sensitization, corneal nerve hypoactivity, and tonic nerve hypoactivity, which causes lacrimal gland dysfunction and is responsible for DED [37,38,40]. Also, inflammatory cytokines interleukin 1, 6, and 8 (IL-1, IL-6, and IL-8) elevated in FM are responsible for hyperosmolarity of lacrimal secretion, leading to apoptosis of corneal epithelial and goblet cells and decreased mucus secretion, resulting in DED, corneal damage, and ocular pain [40,41]. In our study, we used objective tests to assess tear fluid quantity and quality. We used both the Schirmer’s test and the TBUT test, and our data confirm the previous findings that FM patients do have dry eyes more frequently than healthy controls.

This study has several limitations: we included only female patients, although FM is also present in males. This is a monocentric study, and we had a small number of patients; it would be useful to analyze whether the conclusions of this work are generalized. Additionally, we did not collect disease duration, pain scores, or the Fibromyalgia Impact Questionnaire (FIQ) score, and therefore could not assess or adjust for FM severity; patients with different stages and duration of FM were included as a single group. Because these variables may independently influence both ocular surface findings and RNFL measurements, their absence should be considered when interpreting our results. In addition, after Bonferroni correction for the four RNFL quadrant comparisons, the difference in the superior temporal quadrant did not remain statistically significant (corrected *p* = 0.180); nevertheless, RNFL thickness in this quadrant was directionally lower in the FM group, consistent with the pattern reported in previous studies, and this finding should be interpreted with caution given the number of comparisons performed.

Our study’s strength is that we investigated both nerve fiber layer changes and dry eye disease within the same cohort; both have been separately proposed to relate to small-fiber neuropathy in FM, although this study did not include direct measurements of corneal nerve density or peripheral neuropathy to confirm a shared underlying mechanism. To our knowledge, no previous study has combined anterior and posterior segment ocular assessments in FM patients within a single cohort, and our findings support the presence of both types of ocular involvement, whether or not they share a common pathophysiological pathway. Future studies incorporating validated FM severity assessments (e.g., the Fibromyalgia Impact Questionnaire), OCT angiography, ganglion cell complex analysis—which may be more sensitive than peripapillary RNFL measurements in detecting early neurodegenerative changes—corneal confocal microscopy to directly visualize corneal nerve fibers, and longitudinal follow-up are needed to provide a more comprehensive assessment of neuro-ophthalmic involvement in fibromyalgia.

## 5. Conclusions

In conclusion, the present study suggests that patients with fibromyalgia may exhibit ocular alterations involving both the anterior and posterior segments of the eye. The dry eye findings—reduced tear production and impaired tear film stability—were consistent and statistically robust across measures. In contrast, the retinal finding was more limited: RNFL thinning was nominally significant in only one of four quadrants (superior temporal) before correction for multiple comparisons and did not remain significant after Bonferroni adjustment; this isolated finding should be considered exploratory and hypothesis-generating rather than definitive evidence of retinal neurodegeneration in fibromyalgia. These findings may represent objective ocular manifestations associated with fibromyalgia rather than diagnostic markers of the disease. Further multicenter and longitudinal studies with larger cohorts, adjustment for multiple comparisons, and disease severity stratification are needed to clarify the clinical relevance of these parameters and to determine whether they may have potential value as adjunctive biomarkers in the future.

## Figures and Tables

**Table 1:** Demographic characteristics and dry eye test results of the study groups.

	Group 1 (*n* = 42) Mean ± SD	Group 2 (*n* = 37) Mean ± SD	*p*
Age (years)	40.81 ± 5.25	39.03 ± 9.59	0.100
Schirmer test (mm)	8.2 ± 4.36	17.51 ± 2.41	**<0.001**
TBUT (sec)	4.95 ± 2.26	9.86 ± 1.64	**<0.001**

SD: standard deviation; Group 1: patients with fibromyalgia; Group 2: healthy controls. Data are presented as mean ± SD. Mann–Whitney U test was used for group comparisons. *p* < 0.05 was considered statistically significant. Significant *p*-values are shown in bold.

**Table 2:** RNFL thickness values in all quadrants.

RNFL (μm)	Group 1 (*n* = 42) Mean ± SD	Group 2 (*n* = 37) Mean ± SD	*p*	Cohen’s d (95% CI)	*p* (Bonferroni × 4)
Inferior Temporal	123.67 ± 15.51	127.27 ± 15.35	0.304 ^2^	−0.233 (−0.677, 0.210)	1.000
Superior Temporal	117.98 ± 13.98	124.35 ± 13.71	**0.045 ^2^**	−0.460 (−0.908, −0.012)	0.180
Inferior Nasal	72.36 ± 9.81	72.05 ± 9.06	0.875 ^1^	0.033 (−0.409, 0.475)	1.000
Superior Nasal	65.4 ± 11.92	66.92 ± 10.24	0.452 ^1^	−0.136 (−0.579, 0.306)	1.000

SD: standard deviation; RNFL: retinal nerve fiber layer; Group 1: patients with fibromyalgia; Group 2: healthy controls. Data are presented as mean ± SD. ^1^ Mann–Whitney U test; ^2^ Independent *t*-Test. A *p*-value of <0.05 was considered statistically significant. Statistically significant *p*-values are shown in bold. CI: confidence interval. Cohen’s d represents the standardized mean difference (Group 1 − Group 2), calculated using the pooled standard deviation; negative values indicate lower RNFL thickness in the FM group relative to controls. Bonferroni-corrected *p*-values account for the four RNFL quadrant comparisons.

**Table 3:** Correlation between tear film parameters and RNFL thickness in the FM group.

RNFL (μm)	Schirmer Test, r (*p*)	TBUT, r (*p*)
Inferior Temporal	0.222 (0.297)	0.059 (0.769)
Superior Temporal	−0.059 (0.784)	−0.108 (0.591)
Inferior Nasal	0.163 (0.447)	0.273 (0.168)
Superior Nasal	−0.099 (0.644)	−0.231 (0.247)

r: Pearson correlation coefficient. Schirmer test correlations based on *n* = 24 FM patients with complete Schirmer and RNFL data; TBUT correlations based on *n* = 27 FM patients with complete TBUT and RNFL data. No correlation reached statistical significance (*p* < 0.05).

## Data Availability

The data presented in this study are available on request from the corresponding author due to privacy and ethical restrictions.

## Abbreviations

The following abbreviations are used in this manuscript:

ACR	American College of Rheumatology
DED	Dry eye disease
FM	Fibromyalgia
OCT	Optical coherence tomography
RNFL	Retinal nerve fiber layer
TBUT	Tear film breakup time
